# Evaluating the predictive performance of gut microbiota for the early-stage colorectal cancer

**DOI:** 10.1186/s12876-022-02599-x

**Published:** 2022-12-12

**Authors:** Maedeh Amini, Sama Rezasoltani, Mohamad Amin Pourhoseingholi, Hamid Asadzadeh Aghdaei, Mohammad Reza Zali

**Affiliations:** 1 Basic and Molecular Epidemiology of Gastrointestinal Disorders Research Center, Research Institute for Gastroenterology and Liver Diseases, Shahid Beheshti University of Medical Sciences, Tehran, Iran; 2 Section Mass Spectrometry and Proteomics, Institute of Clinical Chemistry and Laboratory Medicine, University Medical Center Hamburg-Eppendorf (UKE), Hamburg, Germany; 3 Gastroenterology and Liver Diseases Research Center, Research Institute for Gastroenterology and Liver Diseases, Shahid Beheshti University of Medical Sciences, Tehran, Iran

**Keywords:** Colorectal cancer, Intestinal microbiota, *F. nucleatum*, *S. bovis*, Bayesian approach, Receiver operator characteristic curve, Latent class analysis

## Abstract

**Background:**

Colorectal cancer (CRC) has been regarded as one of the most frequently diagnosed malignancies among the leading causes of cancer-related morbidity and mortality globally. Diagnosis of CRC at the early-stages of tumour might improve the survival rate of patients. The current study sought to determine the performance of fecal *Fusobacterium nucleatum* (*F. nucleatum*) and *Streptococcus bovis* (*S. bovis*) for timely predicting CRC.

**Methods:**

Through a case–control study, the fecal sample information of 83 individuals (38 females, 45 males) referring to a hospital in Tehran, Iran was used. All patients underwent a complete colonoscopy, regarded as a gold standard test. Bacterial species including *S. bovis* and *F. nucleatum* were measured by absolute quantitative real-time PCR. The Bayesian univariate and bivariate latent class models (LCMs) were applied to estimate the ability of the candidate bacterial markers in order to early detection of patients with CRC.

**Results:**

Bayesian univariate LCMs demonstrated that the sensitivities of *S. bovis* and *F*. *nucleatum* were estimated to be 86% [95% credible interval (CrI) 0.82–0.91] and 82% (95% CrI 0.75–0.88); while specificities were 84% (95% CrI 0.78–0.89) and 80% (95% CrI 0.73–0.87), respectively. Moreover, the area under the receiver operating characteristic curves (AUCs) were 0.88 (95% CrI 0.83–0.94) and 0.80 (95% CrI 0.73–0.85) respectively for *S. bovis* and *F*. *nucleatum*. Based on the Bayesian bivariate LCMs, the sensitivities of *S. bovis* and *F. nucleatum* were calculated as 93% (95% CrI 0.84–0.98) and 90% (95% CrI 0.85–0.97), the specificities were 88% (95% CrI 0.78–0.93) and 87% (95% CrI 0.79–0.94); and the AUCs were 0.91 (95% CrI 0.83–0.99) and 0.88(95% CrI 0.81–0.96), respectively.

**Conclusions:**

Our data has identified that according to the Bayesian bivariate LCM, *S. bovis* and *F. nucleatum* had a more significant predictive accuracy compared with the univariate model. In summary, these intestinal bacteria have been highlighted as novel tools for early-stage CRC diagnosis.

## Background

Colorectal cancer (CRC) as one of the commonest malignancies, excludes from the glandular, epithelial cells of the large intestine. The cancer appears when certain cells of the epithelium get a series of genetic or epigenetic mutations [1]. CRC remains the second leading cause of cancer-related mortality and has been ranked as the 16th leading cause of death among all diseases and injuries in the entire world. Moreover, the absolute numbers of incidence and mortality cases of CRC have incremented in Asia, America, and Europe as well as worldwide [2, 3]. The considerable global burden of CRC can be attributed to side effects of treatment, medical costs, and using health care services [4]. More surprisingly, the increasing CRC mortality in some parts of the world indicates that early diagnosis rate was low [1, 5]. Whilst early detection of patients with CRC is an effective manner to prolong life and subsequently plays a key role in improving the 5-year survival rate [6]. The mean five-year survival rate for those with the earliest stage (stage I) can be high as 90%, while for CRC patients with the advanced stage (stages III and IV), it can be less than 10% [7]. Generally, the burden of CRC highlights the requirement for more efficient interventions in terms of primary prevention. Meanwhile, early diagnosis and treatment of CRC has emerged as a vitally important global topic to improve the survival rate of patients [8].

Since at early-stage, there are no clinical symptoms in most patients suffered from CRC, timely detection is mainly achieved through screening the asymptomatic individuals [9]. Based on this, multiple screening modalities for CRC is recommended by most guidelines including fecal tests (e.g. guaiac-based fecal occult blood testing (FOBT) and fecal immunochemical test (FIT) and colorectal endoscopy (e.g. colonoscopy and/or flexible sigmoidoscopy) [10, 11]. Nevertheless, each one of the tests has its own merits and drawbacks. The colonoscopy is considered by many to be the gold standard (GS) of screenings because it provides early detection as well as effective removal of preneoplastic lesions. Further, this test has high sensitivity and specificity for detecting adenomas and CRC. Nonetheless, colonoscopy is an invasive test, requires repeating frequently (3–5 years), is expensive to implement, and has poor compliance rates. These limitations make this test unsuccessful as a screening instrument in some countries [12]. Hence, a drive to develop highly accurate screening methods has stimulated substantial interest in investigating potential biomarkers for people who are unwilling to participate in colonoscopy examination in order to early detection of patients with CRC.

Research results from early evidence suggested a role for microorganisms in CRC development [13]. Increased attention has been paid to the effect of gut microbiota in the initiation and progression of CRC. Accordingly, numerous papers and reviews proved that gut microbiome (microbiota) may influence tumour development via the virulence factors of the pathogenic bacteria [14–17]. In this context, accumulating evidence has revealed alternations in several bacterial species such as *Fusobacterium nucleatum* (*F. nucleatum*) with a potential impact on mucosal immune response which were significantly elevated in stool specimens from CRC patients than the healthy control group [18, 19]. Another potential novel bacterial marker is *Streptococcus bovis* (*S. bovis*) which its association with cancer is well described in the literature. *S. bovis*, a normal inhabitant of the human gastrointestinal tract, might cause bacteremia, endocarditis, and urinary infection. Some of the studies have identified that there is a correlation between *S. bovis* presence in stool and colorectal neoplasia [20, 21]. In addition to the strong clinical correlation, recent published articles have declared that colon cancer development is promoted actively by *S. bovis* via *β*-catenin signalling pathway. These results support that although *S. bovis* has a strong correlation with CRC, it is functionally included in the development of CRC and may also has a causal role in CRC [22, 23].

An active field in biomedical and statistical research is evaluating the predictive performance of different types of biomarkers through the computation of some classification accuracy measurements such as sensitivity, specificity, and area under the curve (AUC) at various cut-points of the diagnostic biomarker’s outcomes. For estimating the accuracy measurements, the biomarker results are compared with the results of the GS. In some situations, the true disease status of participants is not known, because of the GS test problems (e.g., invasive, expensive, risk of complications) [24]. In this case, calculation of diagnostic accuracy measures is likely to be sophisticated. To overcome no gold standard situations in diagnostic accuracy research, different statistical techniques have been proposed. As reported by large studies, latent class models (LCMs) have been increasingly utilized to assess the accuracy of diagnostic tests in which it is not assumed that the test is perfect [25]. In brief, latent class refers to the fact that the true disease status of the individual is hidden and probabilistic estimates can be made for establishing this situation [26].

Since colonoscopy as a perfect reference standard test does have some disadvantages, we would like to examine whether *S. bovis* and *F. nucleatum* can be good predictors for CRC in the absence of the GS test results. Previous research studies have already demonstrated an association between the bacterial markers and CRC [27, 28]. However, few literatures have focused on assessing the diagnostic efficacy of the two markers to correctly recognize patients at risk of CRC. So currently, the main objective of our work was to explore the ability of *F. nucleatum* and *S. bovis* for early identification of CRC using Bayesian LCM in a sample of Iranian population, when the results of the GS test were not known. In CRC, as the best of our knowledge, a Bayesian latent class analysis for evaluating the predictive power of *F. nucleatum* and *S. bovis* has never been applied across the globe. Notably, the results from the absence of gold standard test were compared with those from the presence of gold standard test.

## Methods

### Study population and clinical procedure

This was a retrospective case–control study which was conducted in Taleghani Hospital affiliated to Shahid Beheshti University of Medical Sciences in Tehran, Iran. A sample of 83 subjects was recruited from June 2016 to December 2018. The participants were chosen according to the random sampling approach. An organized questionnaire was applied to collect information from study subjects. Total fresh stool samples were collected 24 h before colonoscopy and bowel cleansing procedures associated with the routine screen. All colonic biopsy samples were classified after colonoscopy and confirmed by an expert pathologist. Patients were consisted in the current study if they met the following criteria: having symptoms namely rectal bleeding, change of bowel habit, anemia, and abdominal pain among patients underwent colonoscopy for screening. Patients were excluded if they met any of the following criteria: (a) using antibiotics, prebiotics and probiotics in the last six months; (b) having a vegetarian diet; (c) performing a medical intervention such as endosonography, endoscopy, endoscopic retrograde cholangiopancreatography and sphincterotomy in the last three months; (e) having history of any cancer, inflammatory or infectious diseases of the intestine; (f) having other gastrointestinal complaints, including Crohn's, inflammatory bowel disease, irritable bowel syndrome, ulcerative colitis, liver disorder, and non-alcoholic fatty liver disease.

Participants were given stool collection containers with a stabilization buffer (0.5 mol/L Tris, 0.15 mol/L EDTA and 10 mmol/L NaCl, pH 9.0) and asked to store the samples in their home, in − 20 °C freezer immediately. Frozen samples were then delivered to the Taleghani hospital and stored at − 80 °C immediately, until more analysis. A standard curve was plotted in order to enumerate target bacteria in fecal samples by absolute quantitative real time PCR. Bacterial species, including *S*. bovis/gallolyticus (ATCC 49,147) and *F*. *nucleatum* (ATCC 25,586) were provided by the Namazi Hospital, Shiraz, Milad Hospital, Tehran, and Iran University of Medical Sciences, Tehran, Iran. The 16S rDNA from Roseburia spp. was purchased from cloned 16S rDNA libraries (Nedayefan Company, Tehran, Iran). Anaerobe isolates were cultured on selective media, and the cultures were incubated at 37 °C in an anaerobic chamber for 48 h (Anoxomat: MART Microbiology B.V. the Netherlands, 0% O_2_, 10% CO_2_, 80% N_2_). The media for microorganisms were as follows. Blood agar (Difco, Heidelberg, Germany), for *S*. bovis/gallolyticus and fastidious anaerobe broth (LabM), supplemented with 1% glucose for *F*. *nucleatum*. The whole number of bacterial cells that had been cultured (the number of colony-forming unit (CFU) was counted with a Neubauer chamber, three times independently by three expert individuals. Eight- fold serial dilutions of the bacterial suspension were prepared and the resulting dilutions were independently counted. DNA was extracted from each different serial dilution of bacterial culture and the concentration was presented as CFU (101–108), for plotting standard curves and counting target bacteria in fecal samples. The oligonucleotide primers were designed for *S*. bovis through the primer express software to qPCR recommendations (Applied Biosystems, CA, USA). For *F*. *nucleatum*, the primers were selected from published specific primers. All pairs of primers were tested for their specificity, using the NCBI BLAST tool. The real time PCR was performed, using ABI 7500 (applied Biosystem). The reaction mixture included SYBR Premix EX Taq II (2 ×) (TLi RNaseH Plus), 20 pmol of forward and reverse primer and 2 ϻl of extracted DNA. [17].

### Statistical analysis

Initially, it was summarized the demographic and clinical characteristic of the study population. Data were expressed as mean ± standard deviation (SD) for continuous variables and number (percentage) for categorical variables. To test whether distributions of bacterial markers deviate from normality, it was used a Kolmogorov–Smirnov test as an overall test of normality as well as specific tests of skewness and kurtosis. Statistical difference between the patients and control groups was compared using the independent sample t-test for normally distributed data or the Mann–Whitney U-test for the non-normal distributed dataset. The significance level was considered to be *p* < 0.05 and IBM SPSS Statistics for windows, version 26.0 (IBM Crop., Armonk, NY, USA) was employed for all descriptive analyses.

In the next section, the predictive powers of *F. nucleatum* and *S. bovis* individually for CRC, were estimated performing Bayesian univariate and bivariate latent class analyses which is described as follows. At first, it was supposed that the true disease status is unknown. It means that in our datasets, the outcome of colonoscopy as a perfect reference standard test, is not obtained in all patients. Thus, the accuracy of *F. nucleatum* and *S. bovis* for early detection of CRC could be determined by LCM in which each marker is imperfect in identifying the true disease status. Basically, in LCM, the true disease status of an individual is considered as a latent variable, *D*, with two mutually exclusive categories (Diseased and Non-diseased). The manifest continuous variables, *Y*_1_, *Y*_2_, …, *Y*_*k*_, that express the *k* diagnostic tests outcomes, give an indication on disease status. Now, let *Y*_*ki*_ (*k* = 1, 2) denote the result of the considered intestinal bacteria for the *i*th individual (*i* = 1, …, *n*) and *D*_*i*_ be the binary latent disease status for individual *i* where *D* = 1 indicates a subject from the diseased population and *D* = 0 denotes a subject from the non-diseased population. It is assumed that observations *i* = 1, …, *n* are a random sample generated from normal distribution according to the following hierarchical model

1 $$D_{i} \sim Bernoulli\left( {\pi_{i} } \right),\;\;\;\;\left( {i = 1, \ldots ,n} \right)$$

$$Y_{i} = \left( {\begin{array}{*{20}c} {Y_{1i} } \\ {Y_{2i} } \\ \end{array} } \right)\sim \varphi_{1} (.|\mu_{D1} , \mu_{D2} , \sigma_{D11}^{2} ,\sigma_{D22}^{2} ,\rho_{D} )^{{D_{i} }} \varphi_{2} (.|\mu_{{\overline{D}1}} , \mu_{{\overline{D}2}} , \sigma_{{\overline{D}11}}^{2} ,\sigma_{{\overline{D}22}}^{2} ,\rho_{{\overline{D}}} )^{{1 - D_{i} }}$$,where $\pi_{i}$ is the probability of a disease such that $P\left( {D_{i} = 1} \right) = 1 {-} P\left( {D_{i} = 0} \right) = \pi_{i}$. $\varphi_{1}$ and $\varphi_{2}$ are the normal probability density function for *F. nucleatum* and *S. bovis* in diseased ($D$) and non-diseased ($\overline{D}$) populations, respectively, and $\mu_{D}$ and $\mu_{{\overline{D}}}$ are the means, and $\sigma_{D}^{2}$ and $\sigma_{{\overline{D}}}^{2}$ are the variances. Further, $\rho_{D}$ and $\rho_{{\overline{D}}}$ are also the correlations between the two markers in each category. In this modelling approach, *D* should be estimated at the first level of the model and then the other parameters at the second level need to be estimated. For obtaining the latent status, employing Bayesian approach is becoming more common for this purpose.

Next, if we have information about the true disease status of tested individuals (i.e., outcomes of GS test), the model in Eq. (1) can be modified for the GS case. Let *Y*_1*lD*_ and *Y*_2*lD*_ denote *F. nucleatum* and *S. bovis* values for *l*th person in a random sample of *m* persons who have the disease (*D*) and also let $Y_{{1j\overline{D}}}$ and $Y_{{2j\overline{D}}}$ represent *F. nucleatum* and *S. bovis* values for *j*th person in a random sample of *s* persons who have not the disease ($\overline{D}$). If the markers measure the same biological phenomenon, the results of them often correlated within the diseased and the disease-free populations conditional on disease status. Thus, we have

2 $$Y_{lD} = \left( {\begin{array}{*{20}c} {Y_{1lD} } \\ {Y_{2lD} } \\ \end{array} } \right)\sim N_{1} \left( {\mu_{D} ,{\Sigma }_{D} } \right), l = 1, \ldots ,m,$$

$$Y_{{j\overline{D}}} = \left( {\begin{array}{*{20}c} {Y_{{1j\overline{D}}} } \\ {Y_{{2j\overline{D}}} } \\ \end{array} } \right)\sim N_{2} \left( {\mu_{{\overline{D}}} ,{\Sigma }_{{\overline{D}}} } \right), j = 1, \ldots ,s,$$

$$\mu_{D} = \left( {\begin{array}{*{20}c} {\mu_{D1} } \\ {\mu_{D2} } \\ \end{array} } \right), \mu_{{\overline{D}}} = \left( {\begin{array}{*{20}c} {\mu_{{\overline{D}1}} } \\ {\mu_{{\overline{D}2}} } \\ \end{array} } \right), {\Sigma }_{D} = \left( {\begin{array}{*{20}c} {\sigma_{D11}^{2} } & {\sigma_{D12}^{2} } \\ {\sigma_{D12}^{2} } & {\sigma_{D22}^{2} } \\ \end{array} } \right), {\Sigma }_{{\overline{D}}} = \left( {\begin{array}{*{20}c} {\sigma_{{\overline{D}11}}^{2} } & {\sigma_{{\overline{D}12}}^{2} } \\ {\sigma_{{\overline{D}12}}^{2} } & {\sigma_{{\overline{D}22}}^{2} } \\ \end{array} } \right),$$

in which, the covariance matrices ${\Sigma }_{D}$ and ${\Sigma }_{{\overline{D}}}$ are positive definite. The proposed model by Choi et al., can be formulated based on marginals for *F. nucleatum* outcomes and conditionals for *S. bovis* given *F. nucleatum* outcomes. After estimating the model parameters including $\mu_{D} , , {\Sigma }_{D} , {\Sigma }_{{\overline{D}}} , \rho_{D}$, and $\rho_{{\overline{D}}}$, the diagnostic accuracy measurements will be calculated as follows.

The Receiver operating characteristic (ROC) curve, is a plot of all possible pairs of the false positive rate (1-specificity) and true positive rate (sensitivity) of the test for cut-off values $c \in \left( { - \infty ,\infty } \right)$ given by

$$\left[ {1 - {\Phi }\left( {\frac{{c - \mu_{{\overline{D}k}} }}{{\sqrt {\sigma_{{\overline{D}kk}}^{2} } }}} \right), 1 - {\Phi }\left( {\frac{{c - \mu_{Dk} }}{{\sqrt {\sigma_{Dkk}^{2} } }}} \right)} \right]$$,

in which, ${\Phi }$ is the cumulative distribution function of a standard normal variable. Notably, we also selected cut-offs that reveal a maximum Youden Index criterion (which is equal to the sum of sensitivity and specificity minus 1) in order to obtain a good trade-off between false-positive and false negative decisions for the values of *F. nucleatum* and *S. bovis*. Moreover, the area under the ROC curve (AUC) which is proposed for an efficient summarization, reflects the quality of the biomarker for discrimination in predicting the outcome. The AUC for each of the markers can be calculated based on the normality assumption as

$${\Phi }\left( { - \frac{{\mu_{{\overline{D}k}} - \mu_{Dk} }}{{\sqrt {\sigma_{{\overline{D}kk}}^{2} + \sigma_{Dkk}^{2} } }}} \right)$$.

This criterion ranges from 0.5 (prediction of biomarker is only by chance) and 1.0 (perfect prediction). The overall accuracy of *F. nucleatum* in comparison with *S. bovis* can be determined by the difference in AUC (i.e., AUC_1_ – AUC_2_) [29].

Because the true values of conditional marker outcome probabilities are often not exactly known in advance, applying fixed parameters might be invalid. In this case, the Bayesian approach provides a way to contain expert prior knowledge concerning parameters. On the other hand, Markov chain Monte Carlo (MCMC) applied to sample from the distribution of the model parameters. In this work, the autocorrelation plots and Geweke's statistic were utilized to check the convergence of Markov chains. Also, since no prior information on the parameters is available, non-informative prior distributions were used for all the parameters (i.e., a beta prior for $\pi$, normal priors for all means, gamma priors for all precisions, and uniform priors for correlations) to obtain estimates. The models were fitted by using OpenBUGS 3.2.3 and the R-package R2OpenBUGS was employed as an interface between R 4.2.1 and OpenBUGS (https://cran.r-project.org/web/packages/R2OpenBUGS).

## Results

### Demographic and clinical characteristics

In total, 83 individuals aged 18–92 years participated in the current study. Of these, for 38 (45.7%) women and 45 (54.3%) men, the mean ages were 58.17 ± 14.69 years and 60.44 ± 14.77 years, respectively. *F. nucleatum* was significantly higher in CRC patients than in controls (29.16 ± 3.31 vs. 21.65 ± 5.16, *p* = 0.005). Moreover, no statistically significant difference between the groups was found with respect to the means of *S. bovis* (*p* = 0.76). It is important to note that of 83 participants, 47 (56.6%) subjects were in CRC group and the remaining 36 (43.4%) people were not.

### Bayesian univariate latent class analysis

Initially, the findings of the univariate models were compared in the case of presence and absence of GS test. Table 1 summarizes the posterior means, standard deviations, and corresponding 95% credible intervals (CrIs) of sensitivity, specificity, and AUC along with optimal cut-off points for each of the bacterial markers resulting from Bayesian univariate modeling approaches. From this Table, it is seen that the sensitivity, specificity, and AUC of *S. bovis* for the diagnosis of early-stage CRC were estimated to be 86% (95% CrI 0.82–0.91), 84% (95% CrI 0.78–0.89), and 0.88 (95% CrI 0.83–0.94), respectively in the absence of a GS test. Subsequently, the sensitivity, specificity, and AUC of *F. nucleatum* were estimated as 82% (95% CrI 0.75–0.88), 80% (95% CrI 0.73–0.87), and 0.80 (95% CrI 0.73–0.85), respectively in the absence of the GS test results. By considering the GS test results, the sensitivity, specificity, and AUC of *S. bovis* were estimated to be 88% (95% CrI 0.79–0.95), 84% (95% CrI 0.78–0.90), and 0.87 (95% CrI 0.81–0.93), respectively. Likewise, for *F. nucleatum*, the sensitivity, specificity, and AUC were 84% (95% CrI 0.79–0.91), 81% (95% CrI 0.76–0.88), and 0.80 (95% CrI 0.74–0.86), respectively.

**Table 1.** Estimated accuracy measures of *F*. nucleatum and *S. bovis* based on the Bayesian univariate models in the presence and absence of gold standard test outcomes

		Mean	SD	MC error	2.5%	97.5%
**Absence of GS test**						
*F. nucleatum*						
	Sensitivity	0.82	0.02	0.003	0.75	0.88
	Specificity	0.80	0.06	0.002	0.73	0.87
	AUC	0.80	0.02	0.001	0.73	0.85
	Cut-off	< 11	-	-	-	-
*S. bovis*						
	Sensitivity	0.86	0.01	0.001	0.82	0.91
	Specificity	0.84	0.01	0.001	0.78	0.89
	AUC	0.88	0.03	0.001	0.83	0.94
	Cut-off	< 16	-	-	-	-
**Presence of GS test**						
*F. nucleatum*						
	Sensitivity	0.84	0.03	0.005	0.79	0.91
	Specificity	0.81	0.05	0.008	0.76	0.88
	AUC	0.80	0.01	0.001	0.74	0.86
	Cut-off	< 21	-	-	-	-
*S. bovis*						
	Sensitivity	0.88	0.02	0.004	0.79	0.95
	Specificity	0.84	0.04	0.001	0.78	0.90
	AUC	0.87	0.01	0.001	0.81	0.93
	Cut-off	< 17	-	-	-	-

*SD *Standard deviation; *MC *Monte carlo; *F. nucleatum *Fusobacterium nucleatum; *S. bovis *Streptococcus bovis; *GS *Gold standard; *AUC* Area under the receiver operating characteristic curve

### Bayesian bivariate latent class analysis

**Fig. 1:**
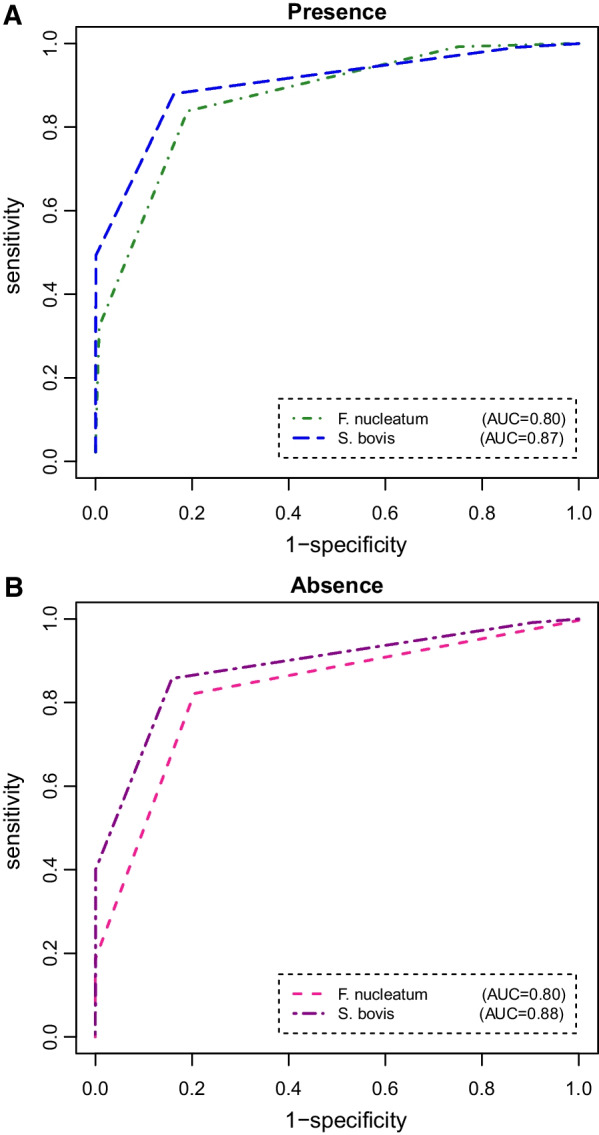
ROC curves estimated for *F. nucleatum* and *S. bovis* from the Bayesian univariate model results in the (**A**) presence and (**B**) absence of gold standard test outcomes

**Fig. 2:**
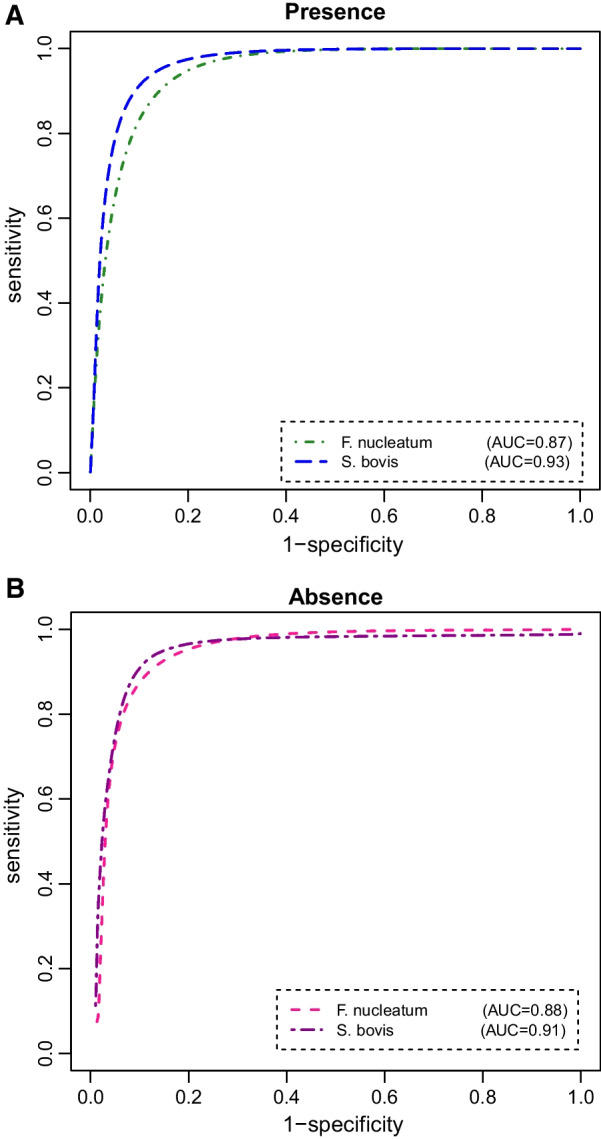
ROC curves estimated for *F. nucleatum* and *S. bovis* from the Bayesian bivariate model results in the (**A**) presence and (**B**) absence of gold standard test outcomes

To address the second goal of our research, the ability of *S. bovis* was compared with *F. nucleatum* for prediction of CRC via bivariate model in the absence and presence of the perfect reference standard information. The posterior means, standard deviations, and corresponding 95% CrIs of sensitivity, specificity, and AUC for each of the markers resulting from fitting Bayesian bivariate models are presented in Table 2. In addition, the correlations between *S. bovis* and *F. nucleatum* separately for CRC groups and difference between AUCs of these markers are displayed in Table 2. Regarding this, in the case of without GS, the sensitivity, specificity, and AUC of *S. bovis* were calculated as 93% (95% CrI 0.84–0.98), 88% (95% CrI 0.78–0.93), and 0.91 (95% CrI 0.83–0.99), respectively. Moreover, the sensitivity, specificity, and AUC of *F. nucleatum* were estimated as 90% (95% CrI 0.85–0.97), 87% (95% CrI 0.79–0.94), and 0.88 (95% CrI 0.81–0.96), respectively. Meanwhile, the estimated AUC difference between the two markers was − 0.03 with 95% CrI (− 0.27) − 0.16, indicting no significance difference in the AUCs between *F. nucleatum* and *S. bovis* (the interval includes zero). By considering the perfect reference standard test results, the sensitivity, specificity, and AUC of *S. bovis* were 93% (95% CrI 0.80–0.99), 89% (95% CrI 0.73–0.95), and 0.93 (95% CrI 0.84–0.98), respectively. Furthermore, the AUC of *F. nucleatum* was 0.87 (95% CrI 0.78–0.91) with a sensitivity and specificity of 91% (95% CrI 0.85–0.97) and 85% (95% CrI 0.77–0.91), respectively. Notably, it is clear that the 95% CrI of the difference in AUCs excludes zero [95% CrI (− 0.14)−(− 0.04)]. This means that *S. bovis* significantly had a better performance compared with *F. nucleatum* for distinguishing amongst CRC groups (with and without CRC). Overall, with respect to the estimated accuracy measurements from the univariate and bivariate models, one can conclude that the bivariate models provided better results. Remarkably, both models, whether with or without GS test, produced rather similar results for *F. nucleatum* and *S. bovis*. Finally, after estimating the latent variable *D* (i.e., latent status of disease), it was concluded that 56 (67.5%) of all study participants were at risk of CRC. Additionally, non-CRC subjects involved of 27 (32.5%) of 83 participants. The Bayesian ROC curves were plotted separately for *S. bovis* and *F. nucleatum* which have been illustrated in Figs. 1 and 2. Obviously, the curves and the corresponding AUCs show that *S. bovis* has better predictive ability to discriminate CRC from normal subjects than *F. nucleatum*.

**Table 2.** Estimated accuracy measures of *S. bovis* and *F. nucleatum* based on Bayesian bivariate models in the presence and absence of gold standard test outcomes

		Mean	SD	MC error	2.5%	97.5%
**Absence of GS test**						
*F. nucleatum*						
	Sensitivity	0.90	0.11	0.007	0.85	0.97
	Specificity	0.87	0.27	0.001	0.79	0.94
	AUC	0.88	0.08	0.005	0.81	0.96
	Cut-off	<94	-	-	-	-
*S. bovis*						
	Sensitivity	0.93	0.09	0.001	0.84	0.98
	Specificity	0.88	0.33	0.002	0.78	0.93
	AUC	0.91	0.08	0.004	0.83	0.99
	Cut-off	<64	-	-	-	-
$\rho_{{D^{ + } }}$		0.76	0.19	0.03	0.64	0.85
$\rho_{{D^{ - } }}$		0.68	0.10	0.03	0.51	0.73
Difference		- 0.03	0.14	0.008	- 0.27	0.16
**Presence of GS test**						
*F. nucleatum*						
	Sensitivity	0.91	0.07	0.001	0.85	0.97
	Specificity	0.85	0.04	0.006	0.77	0.91
	AUC	0.87	0.01	0.002	0.78	0.91
	Cut-off	< 135	-	-	-	-
*S. bovis*						
	Sensitivity	0.93	0.10	0.003	0.80	0.99
	Specificity	0.89	0.11	0.003	0.73	0.95
	AUC	0.93	0.03	0.001	0.84	0.98
	Cut-off	< 29	-	-	-	-
$\rho_{{D^{ + } }}$		0.69	0.11	0.004	0.58	0.82
$\rho_{{D^{ - } }}$		0.64	0.11	0.003	0.54	0.76
Difference		- 0.07	0.03	0.001	- 0.14	- 0.04

*SD* Standard deviation; *MC* Monte carlo; *F. nucleatum* Fusobacterium nucleatum; *S. bovis* Streptococcus bovis; *GS* Gold standard; *AUC* Area under the receiver operating characteristic curve

## Discussion

Annually, over a million people are diagnosed with CRC and so many researches are trying to find more effective strategy for screening and early detection of CRC. Among series of clinical examinations, colonoscopy has been proposed as a gold standard to determine the prognosis of adenoma of the colon and CRC in many countries. However, with respect to the previous studies, the technique entails substantial risk and/or cost. Thus, there are an increasing number of reports to investigate novel markers for detection of asymptomatic early-stage CRC which would be either efficient or cost-effective [30, 31]. To address this need, we attempted to estimate the accuracy of two well-known intestinal microbiota in the early detection of CRC and select high-risk patients for colonoscopy via Bayesian latent class model regardless of colonoscopy outcomes.

*F. nucleatum*, an opportunistic commensal anaerobe in the oral cavity, has been frequently reported that plays an important role in the initiation of CRC and promoting tumor cell growth [19, 32]. Various related researches have reported that *F.* nucleatum is enriched in both the feces and colonic mucosa of CRC patients [33, 34]. Furthermore, a previous study indicated that *F. nucleatum* promotes chemotherapeutic resistance [18]. Sun et al. [35] declared that *F. nucleatum* potentiate CRC development using toll-like receptor 2 (TLR2)/toll-like receptor 4 (TLR4) signaling and microRNA (miRNA)-21 expression. Wu et al. [36] identified that *F. nucleatum* can induce immune suppression of gut mucosa via suppressing the function of immune cells. Repass et al. [37] in metagenomics and transcriptional analyses showed that the enrichment of *F. nucleatum* is significantly incremented compared with adjacent normal tissues. Yamamura et al. [38] reported that *F. nucleatum* is discovered in 20, 10, and 45% of esophageal, gastric, and CRC tissues, respectively. It was also found that *F. nucleatum* infection in the colon is a risk factor for CRC according to the systematic review and meta-analysis by Janati et al. [39]. In the same line, Grobbee et al. [40] observed that the levels of *F*. nucleatum were higher in CRC patients and those with high-grade dysplasia lesions in comparison with those who had normal mucosa. Another important bacterial marker is *S. bovis* which has been linked to the development of CRC over a long period of time [41]. Corredoira et al. [42] reported the connection of *S. bovis* with colon cancer in patients took into account the presence of premalignant adenomas which are usually considered as early-stage precursors of carcinomas. The cause of this association might be that colonic neoplasia permits the overgrowth or translocation of *S. bovis* such that it is causative of neoplasia. In a study by Gold et al. [43], it was shown that between 25 and 80% of patients with *S. bovis* had colorectal adenomatous polyps, aberrant crypt foci, and extracolonic malignancy. Alike, it was previously stated that *S. bovis* was found to increase the production of inflammatory cytokines in the colonic mucosa of rates which is supposed to lead to the development of CRC [20]. However, in the present study, our data were not consistent with those studies in which the authors indicated that *S. bovis* and *F. nucleatum* were significantly present at higher levels in samples from subjects with CRC than samples from healthy subjects [19, 44]. The difference in these results might be due to small sample size of this study. Hence, further investigation with a larger number of patients is needed.

Although the mechanisms and causalities of relationships between *S. bovis* and *F. nucleatum* with CRC have not been still discovered, an array of prior studies have examined the diagnostic performance of the markers in CRC patients. With regard to this, we established the ROC curves to evaluate the diagnostic value of *S. bovis* and *F. nucleatum* for CRC when the outcomes of GS test (i.e., colonoscopy) is unknown. The results showed the bivariate LCM yielded a higher diagnostic accuracy for both of the intestinal bacteria than the univariate model. According to the bivariate model, *S. bovis* had a better discriminant capability with an AUC of 0.91 than *F. nucleatum* for detecting early CRC. It is noteworthy that 93% of patients with CRC and 88% without CRC could be correctly detected by *S. bovis*. Comparing sensitivity, specificity, and AUC for the two markers in the presence and absence of GS results, we found that the estimates were nearly similar. It suggests that the performance of *S. bovis* and *F. nucleatum* for CRC is almost the same with and without GS test. In this perspective, a number of studies conducted on early prediction of CRC have assessed the accuracy of *S. bovis* and *F. nucleatum* considering the colonoscopy results as GS test. Liang et al. [33] in a large cohort of 439 participants found that *F. nucleatum* had the best performance in discriminating CRC from healthy controls giving an AUC of 0.87. Besides, this intestinal marker can serve as a non-invasive diagnostic method for CRC with a moderate sensitivity of 77.7% and specificity of 79.5%. Peng et al. [32] in a meta-analysis study revealed that *F. nucleatum* with pooled sensitivity of 0.81, specificity of 0.77, and AUC of 0.86 is a valuable marker for CRC diagnosis. In another systematic review and meta-analysis study, it was detected that the pooled sensitivity and specificity of fecal *F. nucleatum* for CRC were 71% and 76%, respectively with the AUC of 0.80. In this respect, the authors concluded that the accuracy of *F. nucleatum* is promising for the diagnosis of colorectal tumor [45]. Furthermore, an available evidence has reported a sensitivity of 72% and a specificity of 91% for *F. nucleatum* which suggests that *F. nucleatum* may serve as a potential prognostic biomarker for early CRC screening [46]. As well, an article recently published consistently declared that *S. bovis* and *F. nucleatum* can distinguish CRC cases from non- CRC controls with a high degree of accuracy [17]. Of importance, existing an agreement between all of the above studies which have confirmed that *S. bovis* and *F. nucleatum* might be powerful markers with high AUC to correctly classify subjects into meaningful subgroups. Interestingly, this is in line with our finding in the presence of GS test. Nevertheless, opposite to our findings, some evidence reported relatively low values for sensitivity and/or specificity of the bacterial markers which may cause missed diagnosis of some cases. Whilst, the improvement in sensitivity and specificity can decline the number of missed diagnoses. In view of this, we believe that the low values may be partly due to some reasons. For instance, the used sample size in some of the studies appears not to be enough that this matter might make the estimated sensitivity and specificity questionable. Additionally, the colonoscopy, which is utilized as the gold standard test in clinical setting, might have errors in measurement. Accordingly, the accuracy of the markers is probably affected by such error and may cause sensitivity and specificity to be estimated with bias. Herein, we have addressed this problem with a novel statistical model in the CRC context, which enables us to provide unbiased estimates for the model parameters. It is worth noting that all of the previous studies have accomplished classical approaches for estimating the accuracy indices in the presence of GS test.

In the current study, we applied bivariate LCM to data, as if the gold standard test information was no available for CRC screening, which allows for the diagnostic tests to be correlated as this will often be the case. According to our results, the discriminatory ability of *S. bovis* and *F. nucleatum* successfully enhanced in bivariate model as compared to the use of univariate model. This finding is likely owing to the bivariate analysis explicitly provide additional information by adding the correlation component to the model. Furthermore, as we expected, *S. bovis* and *F. nucleatum* were correlated in diseased and non-diseased groups. Thus, the application of the bivariate model can be helpful for assessing simultaneously the power of the intestinal microbiota. In our literature review, we have not found any study that compare the classification accuracy in the case of with and without gold standard by taking into account the correlation between biomarker measurements taken on the same individuals through bivariate modelling approach. In this work, we have done it for the first time and could therefore be proposed for future similar studies.

The authors of this paper estimated the model parameters within a Bayesian framework which outputs the full distribution for each of the parameters via the iterations saved by the model. This approach is simple to obtain distributions of additional variables, which are calculated form the parameters. Importantly, using the Bayesian analysis, one can evaluate how well the diagnostic test performs in estimating disease status of each subject. Our findings revealed that almost all of the parameters are estimated by narrower credible intervals; consequently, we can conclude that the estimates have relatively high accuracy. Notably, a vast literature has emphasized the importance of Bayesian method in estimating the accuracy of medical tests in the detection and treatment of disease.

### Strengths and limitations

This study has several noteworthy strengths. A key advantage is that this research consists the histologic classification, location, size, and grade of dysplasia for all cases as well as each participant withstood a complete colonoscopy with full visualization of the colon from rectum to cecum. Another important strength is that we first examined the performance of *S. bovis* and *F. nucleatum* concurrently for early CRC diagnosis using latent class model. The biggest advantage of this modelling approach is obviously the evaluation of diagnostic accuracy of test (s) which made it possible to estimate the precision of diagnostic tests in recognition of disease without considering perfect reference standard test results. On the other hand, the bivariate latent class model not only accounts for dependence across test (s) outcomes, but also identify latent sub-populations in data. Additionally, despite application of different cluster methodologies in various studies is warranted, Bayesian LCM proved to be powerful technique to discriminate between cases and controls. As complementary tools, logistic regressions or discriminant analysis may prove valuable to allocate individuals to class membership. Finally, the advantage of the Bayesian method is twofold: (i) the Bayesian estimates are not sensitive to small sample size; (ii) this approach incorporates the prior information to avoid the non-identifiability. Our research is not without limitations that merit attention when interpreting the results. First of all, due to retrospective design of this study, not all clinical data were available. Also, our study population was limited to a sample of Iranian subjects, so caution should be taken in generalizing our results to other populations. Third, because of the case–control study design, recall bias may be an inherent weakness. Fourth, since the sensitivity of FIT test has limitation and the kits of it are of poor quality in Iran, we did not work on this test. Lastly, owing to relatively small sample size, studies with similar design and larger sample size are recommended in order to confirm or refine our findings.

## Conclusions

In the field of colorectal cancer, the authors of this article presented Bayesian bivariate latent class model which can be useful for simultaneous study of intestinal bacteria in classification of patients when the GS test is encountered with problems. Noticeably, the flexibility inherent in this type of models permits the incorporation of the potential dependence among diagnostic tests. On the whole, we demonstrated that application of the methodology described here to the evaluation of accuracy of *S. bovis* and *F. nucleatum* successfully would improve the early-stage identification of CRC regardless of GS test results. For this reason, we think that this method could be offered to conduct similar prospective screening studies with two biomarkers. In particular, based on the obtained results from the Bayesian bivariate LCM, we have found that *S. bovis* is a promising potential and useful screening marker with high accuracy to select high-risk individuals for colonoscopy in order to definitively CRC diagnosis in clinical settings.

## Data Availability

The datasets generated during and analyzed during the current study are not publicly available due to privacy of the study project but are available from the corresponding authors on reasonable request.

## Abbreviations

CRC: Colorectal cancer
FOBT: Fecal occult blood testing
FIT: Fecal immunochemical test
GS: Gold standard
*F. nucleatum*: *Fusobacterium nucleatum*
*S. bovis*: *Streptococcus bovis*
AUC: Area under the curve
LCM: Latent class model
ROC: Receiver operating characteristic
INSF: Iran National Science Foundation
SD: Standard deviation
MCMC: Markov chain Monte Carlo
CrI: Credible interval
